# Signatures of natural selection may indicate a genetic basis for the beneficial effects of oily fish intake in indigenous people from coastal Ecuador

**DOI:** 10.1093/g3journal/jkaf014

**Published:** 2025-01-28

**Authors:** Débora Y C Brandt, Oscar H Del Brutto, Rasmus Nielsen

**Affiliations:** Department of Integrative Biology, University of California at Berkeley, Berkeley, CA 94720, USA; Department Genetics Evolution and Environment, University College London, Gower Street, London WC1E 6BT, UK; School of Medicine and Research Center, Universidad Espiritu Santo—Ecuador, Samborondón 092301, Ecuador; Department of Integrative Biology, University of California at Berkeley, Berkeley, CA 94720, USA; Department of Statistics, University of California at Berkeley, Berkeley, CA 94720, USA; Center for Computational Biology, University of California at Berkeley, Berkeley 94720, CA, USA; GLOBE Institute, University of Copenhagen, Copenhagen 1350, Denmark

**Keywords:** genomics, Ecuador, indigenous American, selection scan, oily fish intake

## Abstract

Atahualpa is a rural village located in coastal Ecuador, a region that has been inhabited by people as early as 10,000 years ago. The traditional diet of their indigenous inhabitants is rich in oily fish and they have, therefore, served as a model for investigating the beneficial effects of such a diet. However, the genetic background of this population has not been studied. In this study, we sequenced the genomes of Atahualpa residents to look for variants under natural selection, which could mediate the effects of oily fish intake. DNA was extracted from 50 blood samples from randomly selected individuals recruited in the Atahualpa Project Cohort. After applying various filters, we calculated genome-wide genotype likelihoods from 33 samples, and combined data from those samples with data from other populations to investigate how the Atahualpa population is genetically related to these populations. Using selection scans, we identified signals of natural selection that may explain the above-mentioned dietary effects. The genetic ancestry of Atahualpa residents is 94.1% of Indigenous American origin, but is substantially diverged from other indigenous populations in neighboring countries. Significant signatures of natural selection were found in the Atahualpa population, including a broad selection signal around the SUFU gene, which is a repressor of Hedgehog pathway signaling and associated with lipid metabolism, and another signal in the upstream region of LRP1B which encodes low-density lipoprotein (LDL) receptor-related protein 1B. Our selection study reveals genes under selection in the Atahualpa population, which could mediate the beneficial effects of oily fish intake in this population.

## Graphical Abstract

**Graphical Abstract:**
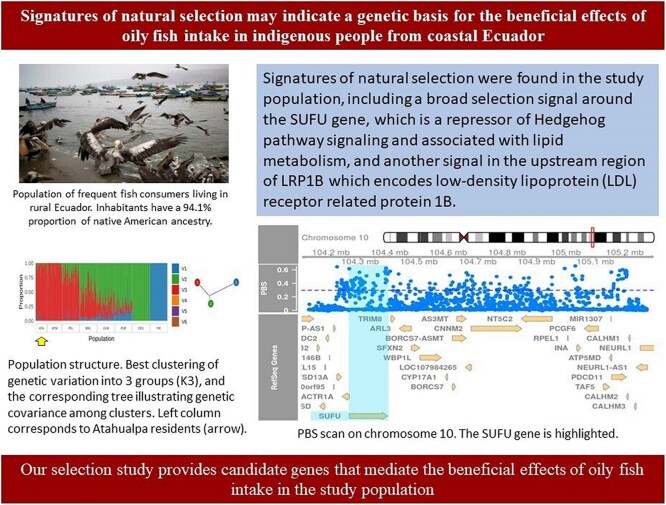

## Introduction

The coastal region of Santa Elena province in Ecuador has long been inhabited by humans. Its location at the extreme West of South America suggests that it could have been home to the first humans of South America, since it is likely that the first people inhabiting this Continent arrived using a Pacific coastal route (Dillehay *et al.* 2008). Early evidence of human settlements in the extreme Southern tip of South America supports the Pacific coastal route hypothesis (Dillehay *et al.* 2008). More concretely, archeological studies in Santa Elena revealed a rich history of human life in the region with evidence of plant (squash) domestication as early as 11k years ago and the presence of diverse cultures, e.g. Las Vegas (8500–4600 B.C.E.) (Raymond 2008) Valdivia (4400–1450 cal B.C.E.), Machalilla (1430–830 cal B.C.E.), Chorrera (1300–300 cal B.C.E.) (Zeidler 2008), Manteño and Huancavilca and the Inca empire (around 1470 C.E.) (McEwan and Delgado-Espinoza 2008). More recently (1532), Spaniards arrived in Ecuador and spread through the country.

Atahualpa is a rural village located in Santa Elena. There is historical evidence that this village was established before the Spanish arrival, and there is little migration to or from the village, which suggests its inhabitants are likely to have a large proportion of indigenous ancestry (Del Brutto and Zambrano 2017). The most recent (2022) Census reports 3,775 inhabitants, 94.3% of whom self-reported as Mestizos, 1.9% Indigenous, 1.7% Montubios, 1.1% Afro-Ecuadorians, 0.6% White and 0.3% as other categories. The previous Census, from 2010, reported 3,532 inhabitants, with 90.3% Mestizos, 4.9% Afro-Ecuadorians, 1.3% Montubios, 1.3% White, 0.5% Indigenous and 1.7% other categories. The proportion of self-reported Mestizos in Atahualpa is higher than in the country as a whole, where 77.5% of the population identify as Mestizos (71.9% in 2010). Although the Mestizo ethnicity suggests admixture, previous studies have shown that people who are self-reported as Mestizos in Ecuador can have a high proportion of indigenous genetic ancestry (Nagar *et al.* 2021).

People from Atahualpa consume high amounts of oily fish as part of their traditional diet, and oily fish intake in this population has been associated with several positive outcomes on their cardiovascular health (Del Brutto, Mera, Gillman, Castillo, *et al*. 2016; Del Brutto, Mera, Gillman, Zambrano *et al*. 2016; Del Brutto *et al.* 2018; Del Brutto, Mera, *et al*. 2021; Del Brutto, Recalde, *et al*. 2021b). Oily fish, and marine animals in general, are rich in omega-3 polyunsaturated fatty acids (PUFAs), which have been implicated in positive cardiovascular effects in several other studies, although not replicated in broad-scale studies (Manson *et al.* 2019).

In another population that consumes a diet extremely rich in omega-3 PUFAs, the Greenland Inuit, previous studies found a strong signature of natural selection in fatty acid desaturase (FADS) genes (Fumagalli *et al.* 2015). Variants of FADS genes present in the Greenland Inuit regulate metabolic pathways to compensate for the high dietary intake of omega-3 PUFAs, which indicates that this population is genetically adapted to this diet (Fumagalli *et al.* 2015). A subsequent study showed that the same genes also had strong signatures of selection in many Native American populations (Amorim *et al.* 2017), although much of that selection appears to have occurred before the peopling of the Americas (Mathieson 2020). This result indicates that the selective pressure on FADS genes could have acted in the ancestors of all Native American populations, during the Upper Paleolithic (Mathieson 2020).

These observations motivated us to search for signatures of natural selection in the people from Atahualpa and investigate whether natural selection has also acted in this population on genes related to fatty acid metabolism. We hypothesize that selection may have acted in response to their traditional diet rich in omega-3 PUFAs, and that selected variants could mediate the beneficial effects of this diet on their cardiovascular health and other outcomes.

Here, we describe the genetic relatedness of the people from Atahualpa to populations from the Americas and other parts of the world. We also perform a genomic scan for natural selection and report several regions that show genetic signatures of selection, including some genes related to fatty acid metabolism.

## Materials and methods

### Ethical approval

Participants of this study were informed and signed a comprehensive informed consent document attesting that they agreed to use their blood samples for DNA extraction and using their anonymized genetic data for research and publications. The Institutional Review Board of Hospital Clínica Kennedy, Guayaquil, Ecuador (FWA: 00030727), approved the study.

### DNA extraction

DNA was extracted from 50 blood samples from randomly selected individuals recruited in the Atahualpa Project cohort (Del Brutto *et al.* 2014). Selection took into account 10 samples from each of the 5 most common last names among Atahualpa residents. Although some level of inbreeding is expected when sampling individuals from a small population, we have ensured that there were no first-degree relatives in this sample. DNA samples were numbered and no identifiable information about these samples was provided to the authors of this study by the Atahualpa Project team.

### Library preparation

Five samples were excluded due to low DNA concentrations in the extractions, and we prepared libraries from the remaining 45 samples for short-read massive parallel sequencing.

Extracted DNA was fragmented using Covaris m220 Focused-ultrasonicator for a target fragment size of 350 to 400 bp. Then, we prepared libraries for 150-bp paired-end sequencing on an Illumina HiSeq 4000 sequencer.

Fragment ends were repaired with NEBNext End Repair Module (Catalog num. E6050): 21.25 μL of DNA extract, 2.5 μL of 10× end repair buffer (E6052) and 1.25 μL of end repair enzyme mix (E6051), with a 20 min incubation at 12C and 15 min at 37C. Next, DNA fragments were purified with MinElute PCR purification kit (5× volume of PB, 2 min centrifugation at 8 g, 700 μL of PE, 2-min centrifugation at 8 g, discard flow-through, centrifuge for 1 min at 8 g, elute DNA with 10 μL EB, 15-min incubation at 37C followed by 2-min centrifugation at 16 g). The eluate containing end-repaired DNA fragments was then directed to adapter ligation using the New England Biolabs quick ligation module (Catalog number E6056) following the product protocol except for the incubation, which was done at 20C for 30 min. Next, another round of purification with MinElute columns was done (10× volume of PB, 2-min centrifugation at 8 g, 700 μL of PE, 2-min centrifugation at 8 g, discard flow-through, centrifuge for 1 min at 8 g, add 25 μL EB, and 15-min incubation at 37C followed by 2-min centrifugation at 16 g).

Next, adapter fill-in was performed with Bst DNA polymerase large fragment (M0275) with a 20-min incubation at 65C and 20 min at 80C. Finally, indexing PCR was done with Invitrogen Platinum Taq DNA Polymerase High Fidelity, for dual indexing with P5 and P7 indices. PCR was performed with an initial 60 s at 94C (60 s), followed by 8 cycles of 30 s at 94C, 30 s at 55C, and 30 s at 68C, and a final period of 5 min at 68C.

The PCR product was then submitted to size selection using AMPure magnetic beads to remove fragments smaller than 150 bp or larger than 1000 bp.

Four samples were excluded from further steps due to low concentrations at the expected library size distribution, measured with BioAnalyzer. The 41 libraries with good concentration at the library target size (350–400 bp) were pooled into 2 pools with 22 and 19 samples each. Each pool was sequenced in 2 lanes for 150 paired-end reads on an Illumina HiSeq 4000 instrument. The final coverage achieved after read processing (see next session) was 1.94539×.

### Read processing

The ends of raw sequencing reads were trimmed for adapter sequences and low-quality bases, and filtered for minimum length after trimming using trimmomatic v. 0.38 with parameters ILLUMINACLIP:TruSeq3-PE.fa:2:30:10 LEADING:3 TRAILING:3 SLIDINGWINDOW:4:15 MINLEN:75.

Next, reads were mapped to the human reference genome downloaded from the 1000 Genomes Projecthttps://ftp-trace.ncbi.nih.gov/1000genomes/ftp/technical/reference/human_g1k_v37.fasta.gz, using bwa mem with default options. Mapped reads were filtered for a maximum edit distance of 7 (taken from the NM tag of sam files, using a custom script NM_filter.py available in https://github.com/deboraycb/atahualpa_genomics). Mapped reads were also filtered for a minimum mapping quality score of 15.

Next, we sorted bam files, added sample and lane tags, and merged reads of the same sample sequenced in different lanes into a single bam file per sample, using samtools (version 1.9, Danecek *et al.* 2021). We marked and removed duplicated reads with picard (version 2.18.4, Picard Toolkit 2019), and remapped reads around potential indels using GATK (version 3.5, DePristo *et al.* 2011) IndelRealigner. We used samtools (version 1.9, Danecek *et al.* 2021) to filter out unmapped reads, reads with an unmapped mate, alignment not primary and reads that failed platformQC (sam flag 4+8+256+512=780). Finally, we recalibrated base quality scores with GATK using dbSNP151 know sites. Eight samples were excluded from further analyses due to average coverage below 0.5×. The remaining 33 samples kept for further analyses had an average coverage of 1.94539X.

### Site filters

We used snpCleaner v2.4.3 https://github.com/tplinderoth/ngsQC/to filter sites for coverage and various types of bias. Mapped reads were pre-filtered for a minimum base quality of 20 and proper pairs of reads using samtools options -Q 20 –rf 2 before generating unfiltered genotype calls for snpCleaner. Sites were then filtered for a minimum of 10 individuals covered by at least 1 read (-k 10 -u 1), showing no excess of heterozygous genotypes on an exact test (-H 1e-6), no strand bias (-S 1e-4), no base quality bias (-b 1e-10), no mapping quality bias (-f 1e-4), and no end distance bias (-e 1e-4). A total of 2,561,742,893 sites passed these filters, including variable and non-variable sites within the sample.

We downloaded genome accessibility at http://ftp.1000genomes.ebi.ac.uk/vol1/ftp/release/20130502/supporting/accessible_genome_masks/20140520.strict_mask.autosomes.bed and mappability http://hgdownload.soe.ucsc.edu/goldenPath/hg19/encodeDCC/wgEncodeMapability/wgEncodeCrgMapabilityAlign100mer.bigWig masks and selected sites that pass those masks (with mappability score ≥ 0.5). The intersection of those sites with the ones that passed the previous filters contains 2,029,003,071 sites.

To analyze data from the Atahualpa population in the context of other populations from the region, we merged our dataset with the dataset from Crawford *et al.* (2017). That dataset contained 59,568,964 sites that passed the accessibility and mappability masks above. The intersection of those sites with the ones that passed our filters contained 58,059,354 sites.

### Genotype likelihood

We performed SNP calling on the 58M sites described above using ANGSD (Korneliussen *et al.* 2014). We calculated genotype likelihoods (GATK method, with -GL 2) from the bam files using ANGSD with parameters -remove_bads 1 -only_proper_pairs 1 -uniqueOnly 1, filtering for minimum mapping quality of 30 and minimum base quality of 20. We output the genotype likelihoods in Beagle format and convert it to vcf using a custom Python script (beagle2vcf_v3.py, available in https://github.com/deboraycb/atahualpa_genomics). We then proceeded to merge this vcf with the dataset from Crawford *et al.* (2017), containing genotype likelihoods calculated with the same filters and methods. The VCF files were merged using BCFtools merge (Danecek *et al.* 2021) for a total of 375 samples, whose locations are shown in Supplementary Fig. 1. After filtering for biallelic SNPs, we obtained a final dataset with 21,423,891 SNPs.

### Population genetics analyses

Since the DNA was sequenced at low coverage, we take advantage of population genetics methods that use genotype likelihoods and thus take into account uncertainty in genotypes in all downstream analyses. We use the program PCAngsd (Meisner and Albrechtsen 2018) for principal component analysis, and the program Ohana (Cheng *et al.* 2017), to infer population structure. These programs required the input to be in Beagle format, which was obtained using VCFtools –BEAGLE-GL option (Danecek *et al.* 2011).

For Ohana, we prepared a subset of the data containing only SNPs with minor allele frequency (MAF) over 0.05. MAF was calculated from the merged VCF file using ANGSD, and the merged VCF files were filtered for sites with MAF > 0.05 using BCFtools (Danecek *et al.* 2021). We ran Ohana 3 times for each value of k, for 1,000 iterations. We report results from the replicate number and iteration number with the best likelihood for each value of k.

We performed $F_{ST}$ calculations and population branch statistic (PBS) selection scan using ANGSD (Fumagalli *et al.* 2013), which estimates $F_{ST}$ from genotype likelihoods based on the Reynolds *et al.* (1983) estimator. PBS for a focal population *i* is given by $PBS_{i}=(T_{i,j}+T_{i,k}\text{–}T_{\;j,k})/2$, where $T_{i,j}=-log(1-F_{STi,j})$ and measures the length of the branch connecting population *i* to populations *j* and *k*, at a position or window of the genome (Yi *et al.* 2010). We used Atahualpa as the focal population, and included the Aymara from Bolivia and Peruvians as the other 2 populations. The selection scan was performed on sliding windows of 50 kb, slid by 10 kb. Candidate peaks were selected as those that had at least 6 windows within the top 0.1% of PBS values. We also performed PBS scans with windows of 1 kb and slide of 500 bp in candidate regions identified in the scan with 50 kb windows, to show more detailed plots of these regions. Plots of selection scan peaks with UCSC RefSeq genes from the human genome assembly version hg19 were generated using R package Gviz version 1.38.4 (Hahne and Ivanek 2016).

## Results

### Population structure

**Fig. 1.**
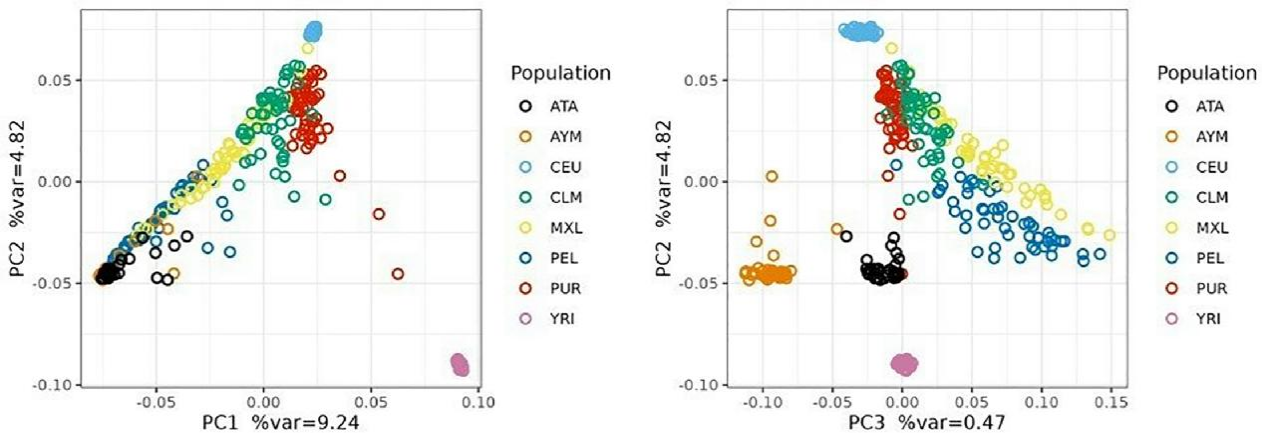
Principal component analysis. Two main axes of variation (PC1 and PC2) show 3 clusters of populations at the extremes of the distribution corresponding to African, European, and Native American ancestries. The third main axis of variation (PC3) separates the people from Atahualpa from other Native American populations (ATA, Atahualpaians; AYM, Aymaras; CEU, Central Europeans; CLM, Colombians; MXL, Mexicans; PEL, Peruvians; PUR, Puerto Ricans; YRI, Yorubas from Nigeria).

**Fig. 2.**
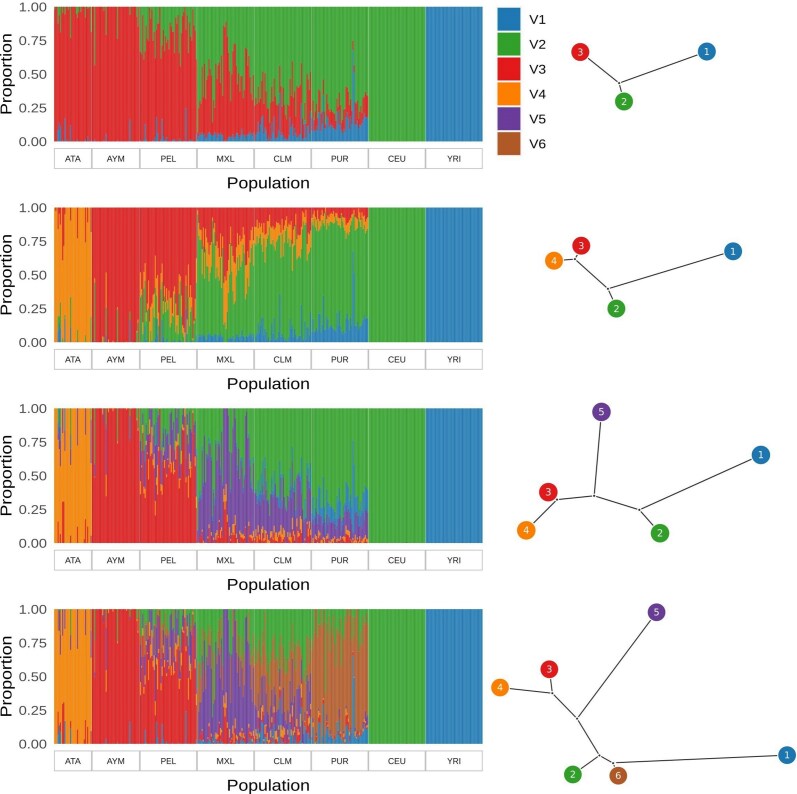
Population structure. Best clustering of genetic variation into 3, 4, 5, and 6 groups, and corresponding trees illustrating genetic covariance among clusters.

Atahualpa samples cluster with other Native Americans in a Principal Component Analysis (PCA) that also includes European and African populations (Fig. 1). The people from Atahualpa are closest to the Aymaras from Bolivia (Crawford *et al.* 2017) and the Peruvians from Lima along the two main axes of genetic variation that together account for 14% of total genetic variation (Fig. 1). Some individuals from Atahualpa are located closer to European (CEU) and African (YRI) samples in the PCA space (Fig. 1), which is evidence of admixture with European and African ancestry components. Evidence of admixture is also observed in structure plots as columns (individual samples) with blue or green sections corresponding to African (YRI) or European (CEU) ancestry components, respectively (Fig. 2).

The Ohana structure results with 3 clusters (*k* = 3) show European and African individuals (CEU and YRI) are best described by a single component each, while the other populations from the Americas are composed of a mixture of those 22 ancestry components and a third component that likely reflects Native American ancestry (Fig. 2). On average, the people from Atahualpa are composed of 94.1% of this Native American ancestry, which is the second highest proportion among our sampled populations, only lower than the Aymara (Table 1).

**Table 1.** Percentage of ancestry components (*k* = 3) reflecting Native American, European, and African ancestry in the populations from the Americas sampled in this study.

Population	Native American (C3)	European (C2)	African (C1)
Atahualpaians	94.1	3.8	2.1
Aymaras	96.6	2.8	0.6
Peruvians	78	19.8	2.2
Mexicans	45.2	50.4	4.4
Colombian	26.1	65.7	8.1
Puerto Ricans	12.8	71.6	15.6

Clustering with 4 components (*k* = 4) splits the Native American component from *k* = 3 into 2. The Atahualpa samples are composed predominantly of one of these Native American sub-ancestries, while Aymara and the other populations from the Americas are predominantly composed of the other Native American sub-ancestry. Increasing the number of clusters to 5 and 6 reveals components that are prevalent in the Mexicans (MXL) and Puerto Ricans (PUR), respectively (Fig. 2).

### Population differentiation and signatures of selection

**Fig. 3.**
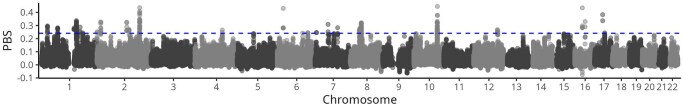
Population branch statistics (PBS) scan for selection in the population from Atahualpa. Dashed line shows 0.1%ile of PBS.

Genome-wide differentiation, measured by $F_{ST}$, is 0.044 between Atahualpa and Aymara, 0.040 between Atahualpa and Peruvians, and 0.016 between Aymara and Peruvians. We use this trio of closely related populations to perform a population branch statistic (PBS) genome-wide scan for natural selection. Sites with high values of PBS demonstrate high genetic differentiation between the focal population (in this case, Atahualpa) and the other 2 populations, which is a signature of natural selection. Figure 3 shows PBS values for 50-kb windows distributed along the genome, sliding by 10 kb. The genome-wide average value of PBS for Atahualpa is 0.034. We identified seven peaks that show more than 6 windows with values of PBS on the 0.1%ile of the genome-wide distribution (Table 2). We describe the candidate genes within those peaks in more detail in Supplementary Material, and we highlight the most striking peaks next.

**Table 2.** Top selection candidate peaks from a PBS scan in the Atahualpa population relative to Aymaras and Peruvians.

Chromosome	Position (Mb)	PBS	Windows
10	105,185	0.441709	14
2	190,915	0.434677	27
1	155,545	0.352242	46
2	16,865	0.331273	9
2	132,665	0.319347	6
8	40.045	0.317198	51
1	25,965	0.298016	7

The scan was performed with windows of 50 kb and slide of 10 kb. Only the windows with the highest PBS values within 1 Mb are listed. Other candidate windows with the 0.1%ile of the genome-wide distribution and within 1 MB of a window with higher PBS value are counted in the column “windows.”

The most striking peak is at position 105 Mb of chromosome 10. Supplementary Fig. 2 zooms into this region and reveals that it is a wide peak spanning almost 1 Mb and includes at least 22 genes. We list the functional information about each of those genes in Supplementary Table 1. We highlight the SUFU gene, which is a repressor of Hedgehog pathway signaling. Activation of the Hedgehog pathway was recently shown to be involved in preventing obesity in adult mice under a high-fat diet (Shi and Long 2017). Knockdown of SUFU led to lower triglyceride levels in Drosophila and decreased the mass of white adipose tissue in mice (Pospisilik *et al.* 2010). Therefore, this gene is clearly involved in fat metabolism and thus could play a role in the positive effects of a diet rich in oily fish on the cardiovascular health of people from the Atahualpa village and related individuals (Del Brutto *et al.* 2018).

There are 3 peaks on chromosome 2: at 191 Mb, 17 Mb, and 133 Mb. The peak at 191 Mb is also wide, spanning approximately 500 kb and overlapping with at least 6 genes (Supplementary Fig. 3). At position 17 Mb, there is a sharper peak upstream of the gene CYRIA (Supplementary Fig. 4). When we zoom into the region at 133 Mb with windows of 1 kb, we find a minor peak with only 3 windows on the gene ANKRD30BL (Supplementary Fig. 5). Next to this region in chromosome 2, at 143 Mb, 2 new peaks arise when we use windows of 1 kb instead of 50 kb (Supplementary Fig. 6). These peaks are upstream of the genes LRP1B and at KYNU.

CYRIA, ANKRD30BL, and KYNU encode proteins with various reported functions, seemingly unrelated to lipid metabolism. CYRIA (CYFIP-Related Rac1 Interactor) encodes a protein predicted to enable small GTPase binding activity and to be involved in the regulation of actin filament polymerization. It is also associated with Synpolydactyly (according to the Gene Summaries from GeneCards, Safran 2010). ANKRD30BL (Ankyrin Repeat Domain 30B Like) does not have functional annotations but it is a paralog of ANKRD30A, which encodes a DNA-binding transcription factor that is uniquely expressed in mammary epithelium and the testis. Changes in expression levels of this gene have been associated with breast cancer progression (according to the Gene Summaries from GeneCards, Safran *et al.* 2010). KYNU (Kynureninase) encodes an enzyme involved in the biosynthesis of NAD cofactors from tryptophan through the kynurenine pathway. Diseases associated with KYNU include Hydroxykynureninuria and Vertebral, Cardiac, Renal, And Limb Defects Syndrome 2. Among its related pathways are superpathway of tryptophan utilization and Kynurenine pathway and links to cell senescence (according to the Gene Summaries from GeneCards, Safran *et al.* 2010). Interestingly, however, LRP1B encodes “low-density lipoprotein (LDL) receptor-related protein 1B,” and variants of this gene have been associated with childhood obesity (Lee 2019). Due to its function, this gene is also a good candidate to mediate the relationship between diet and cardiovascular health in the people from Atahualpa.

Additional relevant peaks of the PBS performed with the trio of populations Atahualpa, Aymara, and Peruvians, were found in the genomewide selection scan, including 2 peaks at chromosome 1: at 155 Mb and 26 Mb. Among the former, we highlighted *FAM189B* (Supplementary Fig. 7), which has been associated with Gaucher disease, a disease that results from a buildup of fatty substances mainly in the liver and spleen. This disease association suggests that this gene could also be a good candidate related to fat metabolism. The peak at 26 Mb of chromosome 1 contains several windows with high PBS values in a narrow region of 100 Kb that is next to 2 genes: *LDLRAP1* and *MAN1C1* (Supplementary Fig. 8). *MAN1C1* is related to the metabolism of proteins and *LDLRAP1* encodes “low-density lipoprotein (LDL) receptor adapter protein 1,” a protein that helps remove cholesterol from the bloodstream. Thus, we also highlighted this peak as a candidate of selection driven by the diet rich in oily fish in the people from Atahualpa.

## Discussion

The people from Atahualpa show a large proportion of Native American ancestry (94.1%), which is even higher than the proportion of Native American ancestry among members of the officially recognized Ecuadorian indigenous group Tsáchila (87.12%) (Nagar *et al.* 2021). Although other populations such as the Aymaras and the Peruvians share a similarly high proportion of Native American ancestry, the population from Atahualpa is genetically differentiated from them, with a distinct ancestry component.

Previous studies have shown a signal of East-West structuring of populations in South America, mainly separating populations from the highlands of the Andes from the populations from the Amazon lowlands (Borda *et al.* 2020; Nakatsuka *et al.* 2020). These recent studies also showed that in the Northern Andes (North of Northern Peru), populations are not as differentiated between East and West as in the Central Andes (the region starting from central Peru and stretching South through Bolivia, Chile, and Argentina). The Northern Andes reach lower altitudes than the Central Andes, and it seems plausible that lower altitudes would allow more gene flow between the coastal region and the Amazon region (Borda *et al.* 2020).

The above-mentioned patterns of population structure along the Andes were described based on Peruvian populations. However, the Ecuadorian highlands are also part of the Northern Andes, and the coastal region of Santa Elena province belongs to a similar dry forest ecoregion as the location of the Tallanes and Moche in Northern Peru, south of the Gulf of Guayaquil. Therefore, it is possible that the genetic component that is almost exclusively present in the Atahualpa population (Fig. 2, k ≥ 4) could be related to the component found in coastal populations from Northern Peru (Tallanes and Moche) (Borda *et al.* 2020). In addition to the proximity and environmental similarity, there is archeological evidence of ancient contact between the people of Northern Peru and Southern Ecuador (Guffroy 2008; Nakatsuka *et al.* 2020). Two possibilities then arise for the origins of this coastal ancestry component: it could be the result of East-West gene flow with Amazonian populations through the Northern Andes or it could be an old component related to the first humans that arrived in South America through the Pacific coast. These possibilities remain to be tested, but the results from Borda *et al.* (2020), who found similarities between populations from the coast and from the Eastern Yunga, suggest the former.

The results from our PBS scan suggest different processes driving the differences in allele frequencies between the people from Atahualpa and 2 closely related populations (Aymara and Peruvians). On one hand, 4 PBS peaks are sharp and indicate the action of selection at specific genes (LRP1B, LDLRAP1, CYRIA, and ANKRD30BL). On the other hand, there are 3 wide peaks (encompassing between 500 Kb and 1 Mb), which contain many genes. The wide PBS peaks make it difficult to pinpoint specific sites that were targets of selection. Nevertheless, the long stretches of divergent sequence in Atahualpa also raise an interesting hypothesis: that these haplotypes were selected in this population after introgression from a diverged population. More specifically, the fact that the PBS signal spans a long sequence [almost 1 Mb in chromosome 10 (Supplementary Fig. 2), approximately 500 kb in chromosome 2 (Supplementary Fig. 3) and approximately 800 kb in chromosome 1 (Supplementary Fig. 7)] indicates a recent process, since the haplotype has not yet been broken by recombination. Further, the fact that the divergence remains high and decreases abruptly at the edges of the block indicates that the haplotype could have been inherited as a whole divergent unit from another relatively distant population. The latter scenario differs from the signature of a haplotype hitchhiking on a new mutation that recently underwent positive selection. In this case, we would expect the signature of high PBS to gradually decrease with distance from the selected mutation. Similarly, we would expect a gradual decrease in PBS if positive selection acted on standing variation that has been segregating many generations before selection started. An alternative scenario for the maintenance of a diverged haplotype with an abrupt decrease in PBS would be that all variants in that haplotype are being maintained by selection, and no linked neutral variation remains (which would generate the pattern of gradual decay in PBS). One such case would be if there was selection maintaining a haplotype in the Atahualpa population, with relaxed selection leading to an accumulation of variants in the other closely related populations.

As previously mentioned, our main motivation for investigating signatures of natural selection in the genomes of the people from Atahualpa came from the observed health benefits associated with the ingestion of oily fish as part of the traditional diet of these individuals. Therefore, we highlight genes present in regions with high PBS scores that have been previously implicated in lipid metabolism. Interestingly, 2 out of the 4 sharp PBS peaks include genes (LRP1B and LDLRAP1) that encode cholesterol receptors. Two other genes (SUFU and FAM189B) also encode proteins that have been associated with lipid metabolism and represent promising candidates of selection within 2 wide peaks of high PBS scores. Therefore, these genes represent a starting point for future investigations to understand the physiological mechanisms that mediate the beneficial effects of diet on the cardiovascular health of the people from Atahualpa and probably in other Indigenous South American populations.

A preliminary report suggested that dietary oily fish intake might be linked to the low prevalence of ischemic heart disease observed in the Inuit (Greenland Eskimos) (Bang *et al.* 1971). Since then, several studies have tried to find an association between intake of this nutrient and lower risk of cardiovascular and other outcomes. However, results have been inconsistent (von Houwelingen *et al.* 1987; Ness *et al.* 1999; Devore *et al.* 2009; van de Rest *et al.* 2009). These contradictory results are possibly related to disparities in study designs or—most importantly—to differences in the characteristics of the study population. In other words, a diet that is beneficial in certain ethnic groups may not have the same effect in others. Fumagalli *et al.* (2015) recovered a signature of selection on FADS genes associated with a diet rich in omega-3 PUFAs in the Inuit. Since Atahualpa residents also have a high dietary intake of oily fish, we investigated whether they show a similar pattern of selection in the FADS genes. However, we found no such signal. Indeed, previous studies have suggested that the selection signal in FADS genes in Native Americans dates back to the Upper Paleolithic, and thus that selection happened in the common ancestor of Inuit and Native Americans (Mathieson 2020). Therefore, it would not be possible to detect a signal of selection in a PBS selection scan comparing different groups from South America, which would not show differential allele frequencies at this locus relative to each other (only relative to populations from outside the Americas).

We also note that while there are dietary similarities between the Atahualpa and the Inuit, their environments differ in many other ways. The Inuit, in their traditional lifestyle, have prolonged exposure to extremely cold weather while the Atahualpa village is located close to the Equator, with a hot climate and 12 daily hours of sunlight exposure all year long. Similarly, the pathogenic environment in the tropics and the Arctic are radically different, and pathogens have been shown to be a major selective pressure driving local adaptation in human populations (Fumagalli *et al.* 2011). These environmental differences may also help explain the differential evolutionary response seen in the Atahualpaians and the Inuit.

Major strengths of this study are the unbiased selection of blood samples including representative individuals who were not first-degree relatives and the use of methods that leverage low coverage sequencing data through the use of genotype likelihoods, which allow merging with other datasets from different populations containing genotype likelihoods calculated with the same filters and methods. Limitations include the sample size, which limits the identification of very low-frequency genetic variants, and the absence of phenotypic data to further validate our candidate genes.

The present study opens new avenues of research by identifying a number of genes associated with lipid metabolism in regions targeted by selection in the Atahualpa genomes, such as the SUFU gene. These are obvious candidate genes for further study in the Atahualpa cohort to corroborate their associations with physiological responses to dietary oily fish intake.

## Supplementary Material

jkaf014_Supplementary_Data

## Data Availability

All study participants signed an informed consent form that reassures them that their data will be stored under safety standards that ensure protection of their privacy and will only be shared with authorized persons. Therefore, the data underlying this study will not be publicly available, but it can be made available upon request, following the conditions and procedures detailed in Supplementary Material. Custom scripts used for genomic data processing are available at https://github.com/deboraycb/atahualpa_genomics. Supplemental material available at G3 online.
