# Supplementary material for: Signatures of natural selection may indicate a genetic basis for the beneficial effects of oily fish intake in indigenous people from coastal Ecuador
Source: G3 (Bethesda). 2025 Jan 28;15(4):jkaf014. doi: 10.1093/g3journal/jkaf014 (PMC12005142; doi:10.1093/g3journal/jkaf014)
Supplement: jkaf014_Supplementary_Data [file jkaf014_supplementary_data.zip › Supplemental_Material_G3-2025-405654.pdf]

# Materials Transfer Agreement (MTA)

## Procedure for requesting access to the full data

Requests for access to the data should be made in writing to the corresponding author and must include the names of the individuals and of their respective employers who will be provided access to the data, the objectives of the research that will be conducted using the data, details on how the results generated by the proposed research will be used, details on what information will be made public in any materials/publications that will be generated from the proposed research, details on how confidentiality of the data that is requested will be guaranteed, and details on all aspects of any intellectual property that may result from the proposed research. All individuals who request access to the data must sign a materials transfer agreement including statements that:

1. They are using the data only for non-commercial research purposes.

2. They will not share the data with anybody else.

3. The data will be stored under conditions that guarantee the confidentiality of human identifiable data

4. They will not attempt to identify individuals

## Parties responsible for evaluating requests

Requests for access to the data will be evaluated by corresponding author Prof. Oscar H. Del Brutto, Prof. Rasmus Nielsen and Dr. Débora Y. C. Brandt.

## Criteria and procedure used to evaluate such requests

The corresponding author will forward the request to the other two authors, who will each evaluate the request based on the conditions listed below and the potential benefit of the proposed research for the Atahualpa population, and provide a written recommendation on approval or disapproval of the request, along with the reasons in the case of disapproval, within 45 days of the request being made. Each author may ask for further clarification on the request before providing a recommendation. In the case of disagreement among the responsible parties, a meeting will be convened to discuss and obtain a consensus decision. If a consensus decision cannot be reached, the final decision will rest with Prof. Oscar H. Del Brutto.

## Description of any conditions for gaining access to the data

1. The requesting party must execute a material transfer agreement provided by the granting parties.

2. Confidentiality of the data provided must be guaranteed, and all liability resulting thereof, by the requesting party in writing, as part of the material transfer agreement. The requesting party must specify how the data will be stored under conditions that guarantee the confidentiality of human identifiable data.

3. The requesting party will not attempt to identify individuals.

4. Results of the research and any public materials/publications that will be released must be shared with the corresponding author for review by the granting parties prior to publication. The granting parties will have 45 days for this review. The requesting party will accommodate all reasonable changes to the materials/publications that are consistent with the conditions outlined herein.

5. Results of the proposed research will not be used nor licensed for commercial purposes.

6. Any intellectual property that is generated by the proposed research will be held jointly by the requesting party and the members of the original research project.

7. The requesting party cannot share the data with anyone that is not listed in the request.

8. The proposed research must be related to cerebral and/or cardiovascular disease, its relationship to diet or drug response, and provide a potential benefit to the studied population.

## Expected response time to requests for data access

Expected response time to requests for data access is 60 days.

## Description of what usage or types of requests would lead to a denial of access

1. Outcomes of the proposed research will be used, patented, or licensed for commercial or university interests by the third party and/or its associates/funders.

2. The third party is not able to guarantee confidentiality of the data that is requested.

3. The proposed research does not provide a potential benefit to the studied population or it is potentially harmful for the studied population.
