# Supplementary material for: Signatures of natural selection may indicate a genetic basis for the beneficial effects of oily fish intake in indigenous people from coastal Ecuador
Source: G3 (Bethesda). 2025 Jan 28;15(4):jkaf014. doi: 10.1093/g3journal/jkaf014 (PMC12005142; doi:10.1093/g3journal/jkaf014)

This file contains supplementary material (Table and Figures) for the study titled "Signatures of natural selection may indicate a genetic basis for the beneficial effects of oily fish intake in indigenous people from coastal Ecuador", by Débora Y. C. Brandt, Oscar H. Del Brutto, and Rasmus Nielsen.

Supplementary Table

Table S1. Candidate genes in chromosome 10, at 105Mb. Gene summaries obtained from GeneCards (Safran et al, GeneCards - the human gene database. Available from: https://www.genecards.org; downloaded on February 1, 2024).

Gene symbol	Gene name	Entrez gene summary
MFSD13A	Major Facilitator Superfamily Domain Containing 13A	Predicted to be integral component of membrane. [provided by Alliance of Genome Resources, Apr 2022]
ACTR1A	Actin Related Protein 1A	This gene encodes a 42.6 kD subunit of dynactin, a macromolecular complex consisting of 10-11 subunits ranging in size from 22 to 150 kD. Dynactin binds to both microtubules and cytoplasmic dynein. It is involved in a diverse array of cellular functions, including ER-to-Golgi transport, the centripetal movement of lysosomes and endosomes, spindle formation, chromosome movement, nuclear positioning, and axonogenesis. This subunit is present in 8-13 copies per dynactin molecule, and is the most abundant molecule in the dynactin complex. It is an actin-related protein, and is approximately 60\% identical at the amino acid level to conventional actin. [provided by RefSeq, Jul 2008]
SUFU	SUFU Negative Regulator of	The Hedgehog signaling pathway plays an important role in early human development. The pathway is a

	Hedgehog Signaling	signaling cascade that plays a role in pattern formation and cellular proliferation during development. This gene encodes a negative regulator of the hedgehog signaling pathway. Defects in this gene are a cause of medulloblastoma. Alternative splicing results in multiple transcript variants [provided by RefSeq, May 2010]
TRIM8	Tripartite Motif Containing 8	This gene encodes a member of the tripartite motif (TRIM) protein family. Based on similarities to other proteins, the encoded protein is suspected to be an E3 ubiquitin-protein ligase. Regulation of this gene may be altered in some cancers. Mutations resulting in a truncated protein product have been observed in early-onset epileptic encephalopathy (EOEE). [provided by RefSeq, Sep 2016]
ARL3	ADP Ribosylation Factor Like GTPase 3	ADP-ribosylation factor-like 3 is a member of the ADP-ribosylation factor family of GTP-binding proteins. ARL3 binds guanine nucleotides but lacks ADP-ribosylation factor activity. [provided by RefSeq, Jul 2008]
SFXN2	Sideroflexin 2	Predicted to enable serine transmembrane transporter activity. Involved in mitochondrial transmembrane transport. Located in mitochondrion. [provided by Alliance of Genome Resources, Apr 2022]
WBP1L	WW Domain Binding Protein 1 Like	Predicted to enable ubiquitin protein ligase binding activity. Predicted to act upstream of or within CXCL12-activated CXCR4 signaling pathway; hemopoiesis; and positive regulation of protein ubiquitination. Predicted to be integral component of membrane. [provided by Alliance of Genome Resources, Apr 2022]
CYP17A1	Cytochrome P450 Family 17 Subfamily A Member 1	This gene encodes a member of the cytochrome P450 superfamily of enzymes. The cytochrome P450 proteins are monooxygenases which

		catalyze many reactions involved in drug metabolism and synthesis of cholesterol, steroids and other lipids. This protein localizes to the endoplasmic reticulum. It has both 17alpha-hydroxylase and 17,20-lyase activities and is a key enzyme in the steroidogenic pathway that produces progestins, mineralocorticoids, glucocorticoids, androgens, and estrogens. Mutations in this gene are associated with isolated steroid-17 alpha-hydroxylase deficiency, 17-alpha-hydroxylase/17,20-lyase deficiency, pseudohermaphroditism, and adrenal hyperplasia. [provided by RefSeq, Jul 2008]
BORCS7	BLOC-1 Related Complex Subunit 7	Part of BORC complex. [provided by Alliance of Genome Resources, Apr 2022]
AS3MT	Arsenite Methyltransferase	AS3MT catalyzes the transfer of a methyl group from S-adenosyl-L-methionine (AdoMet) to trivalent arsenical and may play a role in arsenic metabolism (Lin et al., 2002 [PubMed 11790780]).[supplied by OMIM, Mar 2008]
CNNM2	Cyclin And CBS Domain Divalent Metal Cation Transport Mediator 2	This gene encodes a member of the ancient conserved domain containing protein family. Members of this protein family contain a cyclin box motif and have structural similarity to the cyclins. The encoded protein may play an important role in magnesium homeostasis by mediating the epithelial transport and renal reabsorption of Mg2+. Mutations in this gene are associated with renal hypomagnesemia. Alternatively spliced transcript variants encoding multiple isoforms have been observed for this gene. [provided by RefSeq, Dec 2011]
NT5C2	5'-Nucleotidase, Cytosolic II	This gene encodes a hydrolase that serves as an important role in cellular purine metabolism by acting primarily on inosine 5'-monophosphate and other

		purine nucleotides. [provided by RefSeq, Oct 2011]
RPEL1	Ribulose-5-Phosphate-3-Epimerase Like 1	Predicted to enable metal ion binding activity and ribulose-phosphate 3-epimerase activity. Predicted to be involved in cellular carbohydrate metabolic process; pentose catabolic process; and pentose-phosphate shunt, non-oxidative branch. Predicted to be active in cytosol. [provided by Alliance of Genome Resources, Apr 2022]
INA	Internexin Neuronal Intermediate Filament Protein Alpha	Neurofilaments are type IV intermediate filament heteropolymers composed of light, medium, and heavy chains. Neurofilaments comprise the axoskeleton and they functionally maintain the neuronal caliber. They may also play a role in intracellular transport to axons and dendrites. This gene is a member of the intermediate filament family and is involved in the morphogenesis of neurons. [provided by RefSeq, Jun 2009]
PCGF6	Polycomb Group Ring Finger 6	The protein encoded by this gene contains a RING finger motif, which is most closely related to those of polycomb group (PcG) proteins RNF110/MEL-18 and BMI1. PcG proteins are known to form protein complexes and function as transcription repressors. This protein has been shown to interact with some PcG proteins and act as a transcription repressor. The activity of this protein is found to be regulated by cell cycle dependent phosphorylation. Alternatively spliced transcript variants encoding different isoforms have been identified. [provided by RefSeq, Jul 2008]
TAF5	TATA-Box Binding Protein Associated Factor 5	Initiation of transcription by RNA polymerase II requires the activities of more than 70 polypeptides. The protein that coordinates these activities is transcription factor IID (TFIID). This gene encodes an integral subunit of TFIID associated with all transcriptionally competent forms of

		that complex. This subunit interacts strongly with two TFIID subunits that show similarity to histones H3 and H4, and it may participate in forming a nucleosome-like core in the TFIID complex. Alternative splicing results in multiple transcript variants. [provided by RefSeq, Dec 2015]
ATP5MK, previously ATP5MD	ATP Synthase Membrane Subunit K	Located in mitochondrion. Part of mitochondrial proton-transporting ATP synthase complex. Implicated in mitochondrial complex V (ATP synthase) deficiency nuclear type 6. [provided by Alliance of Genome Resources, Apr 2022]
MIR1307	MicroRNA 1307	microRNAs (miRNAs) are short (20-24 nt) non-coding RNAs that are involved in post-transcriptional regulation of gene expression in multicellular organisms by affecting both the stability and translation of mRNAs. miRNAs are transcribed by RNA polymerase II as part of capped and polyadenylated primary transcripts (pri-miRNAs) that can be either protein-coding or non-coding. [provided by RefSeq, Sep 2009]
PDCD11	Programmed Cell Death 11	PDCD11 is a NF-kappa-B (NFKB1; 164011)-binding protein that colocalizes with U3 RNA (MIM 180710) in the nucleolus and is required for rRNA maturation and generation of 18S rRNA [supplied by OMIM, Oct 2008]
CALHM2	Calcium Homeostasis Modulator Family Member 2	Predicted to enable cation channel activity. Involved in positive regulation of apoptotic process. Predicted to be integral component of plasma membrane. [provided by Alliance of Genome Resources, Apr 2022]

Supplementary Figures

Figure S1. Location of populations included in this study: people from Atahualpa in Santa Elena province (Ecuador) sequenced in this study (ATA), Aymara from Tiwanaku and La Paz (Bolivia) from (Crawford et al. 2017). (AYM), and the following populations from the 1000 Genomes Project (The 1000 Genomes Project Consortium 2015): Colombians from Mendellín (CLM), Peruvians from Lima (PEL), Puerto Ricans from Puerto Rico (PUR), Utah (USA) residents with Northern and Central European ancestry (CEU), Los Angeles (USA) residents with Mexican ancestry (MXL), and Yoruba from Ibadan (Nigeria) (YRI). (Google Maps 2022: https://www.google.com/maps/@18.6090502,-56.5691989,2.92z; downloaded on February 1, 2024).

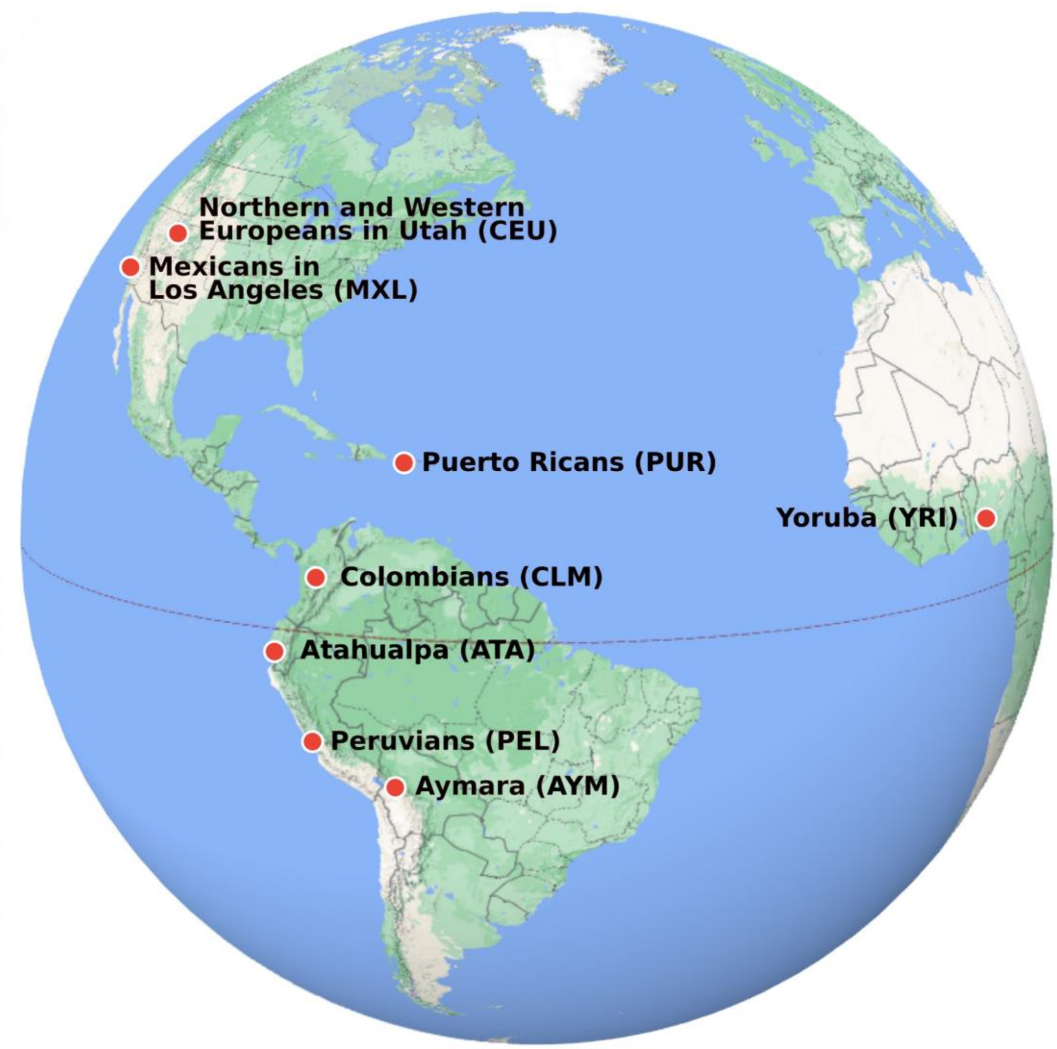

Figure S2. PBS scan peak on chromosome 10. We highlight the SUFU gene, which has been implicated in fat metabolism. Scan performed with windows of 1kb, slide of 500bp. Dashed blue line shows 0.1 percentile of PBS. Black lines show genomewide FST values for each population pair.

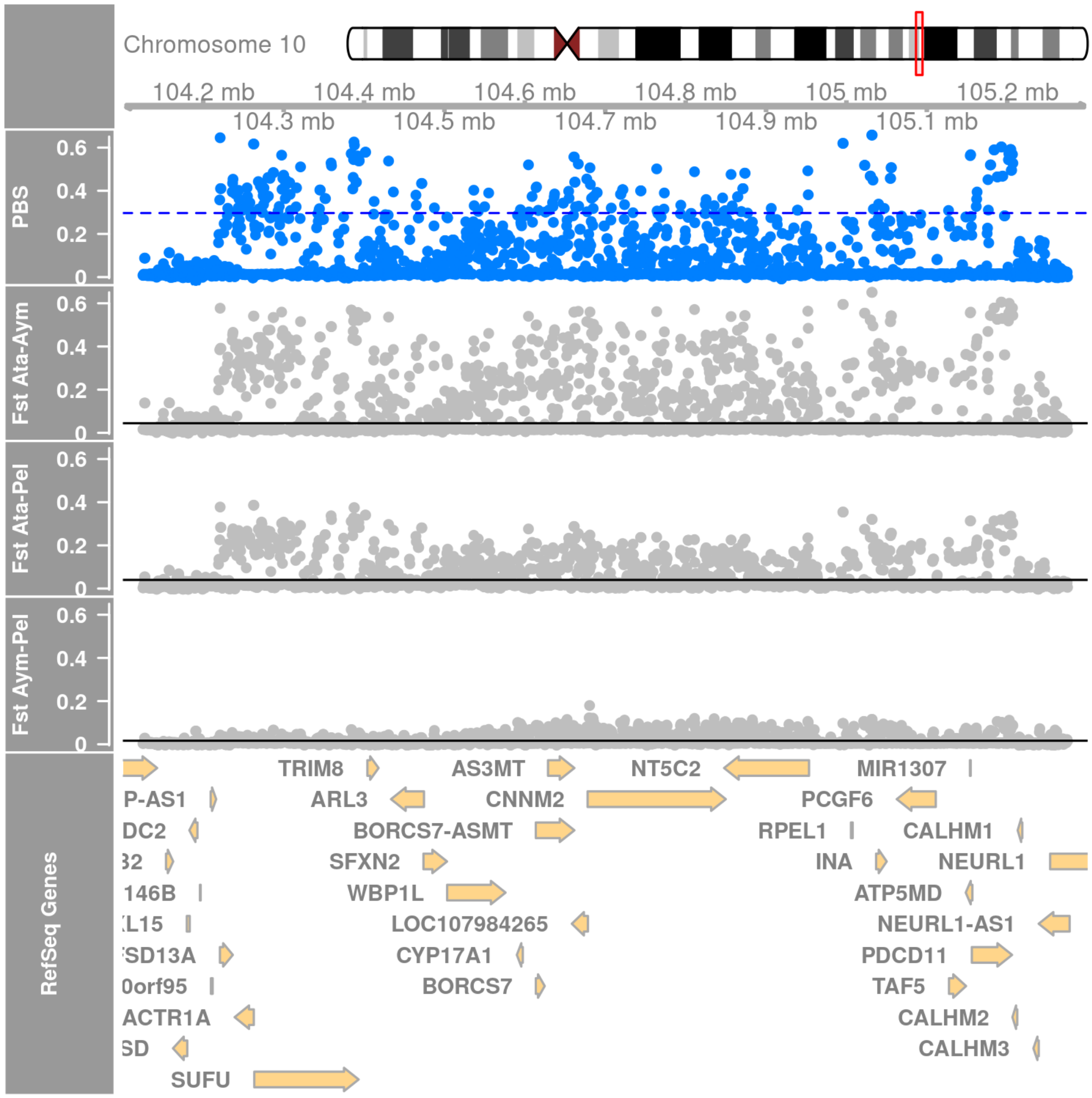

Figure S3. PBS scan peak on chromosome 2 at 190Mb. Scan performed with windows of 1kb, slide of 500bp. Dashed blue line shows 0.1 percentile of PBS. Black lines show genomewide FST values for each population pair.

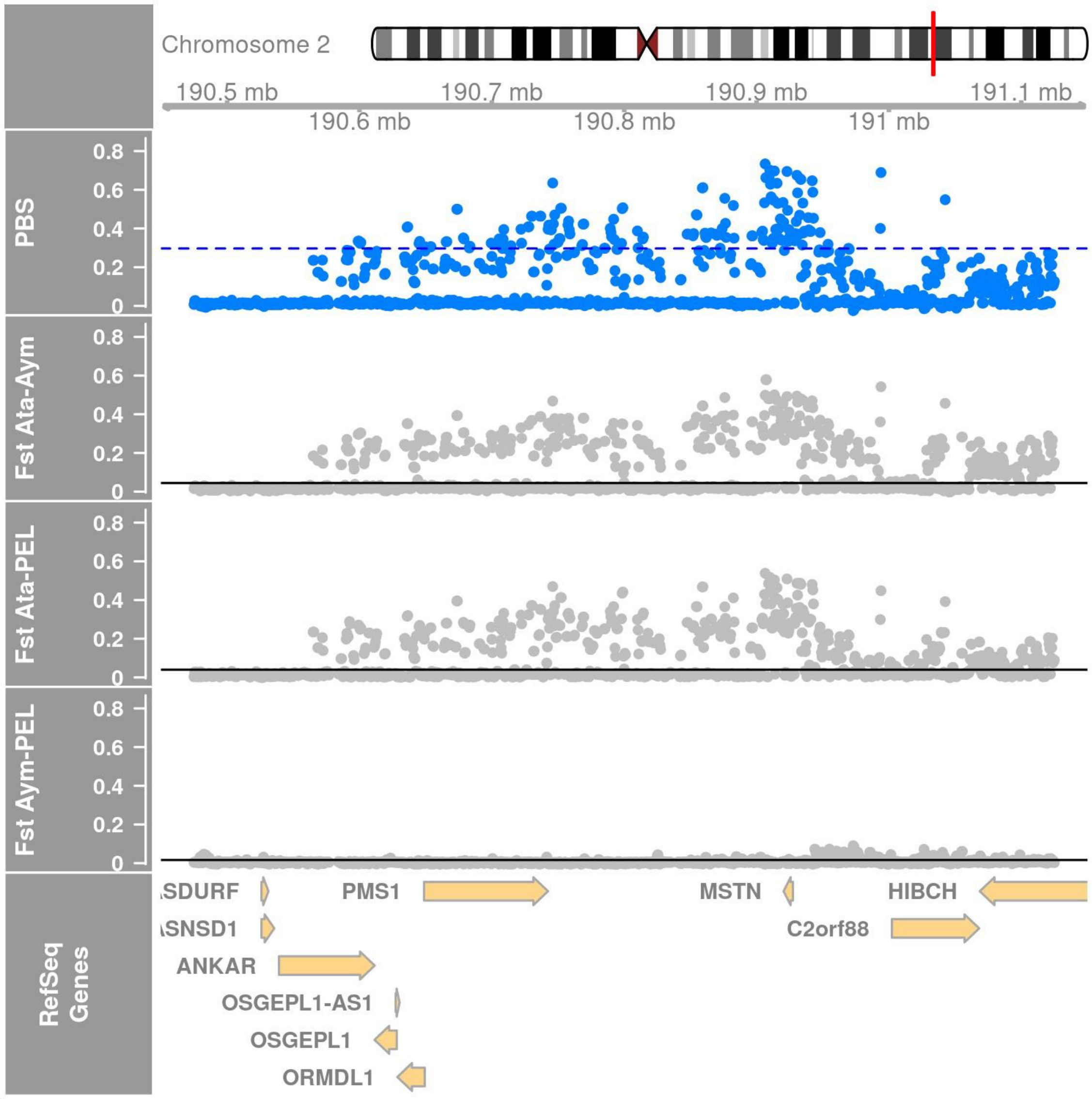

Figure S4. PBS scan peak on chromosome 2 at 17Mb. Scan performed with windows of 1kb, slide of 500bp. Dashed blue line shows 0.1 percentile of PBS. Black lines show genomewide FST values for each population pair.

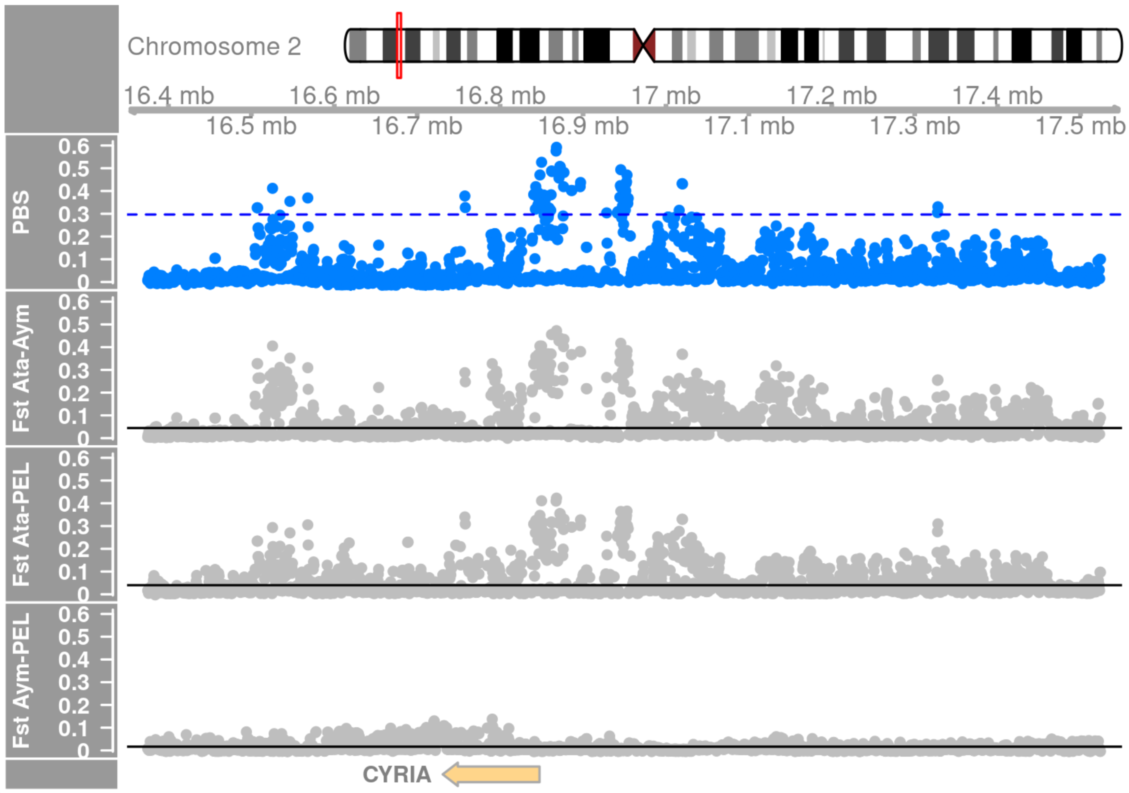

Figure S5. PBS scan peak on chromosome 2 at 133Mb. Scan performed with windows of 1kb, slide of 500bp. Dashed blue line shows 0.1 percentile of PBS. Black lines show genomewide FST values for each population pair.

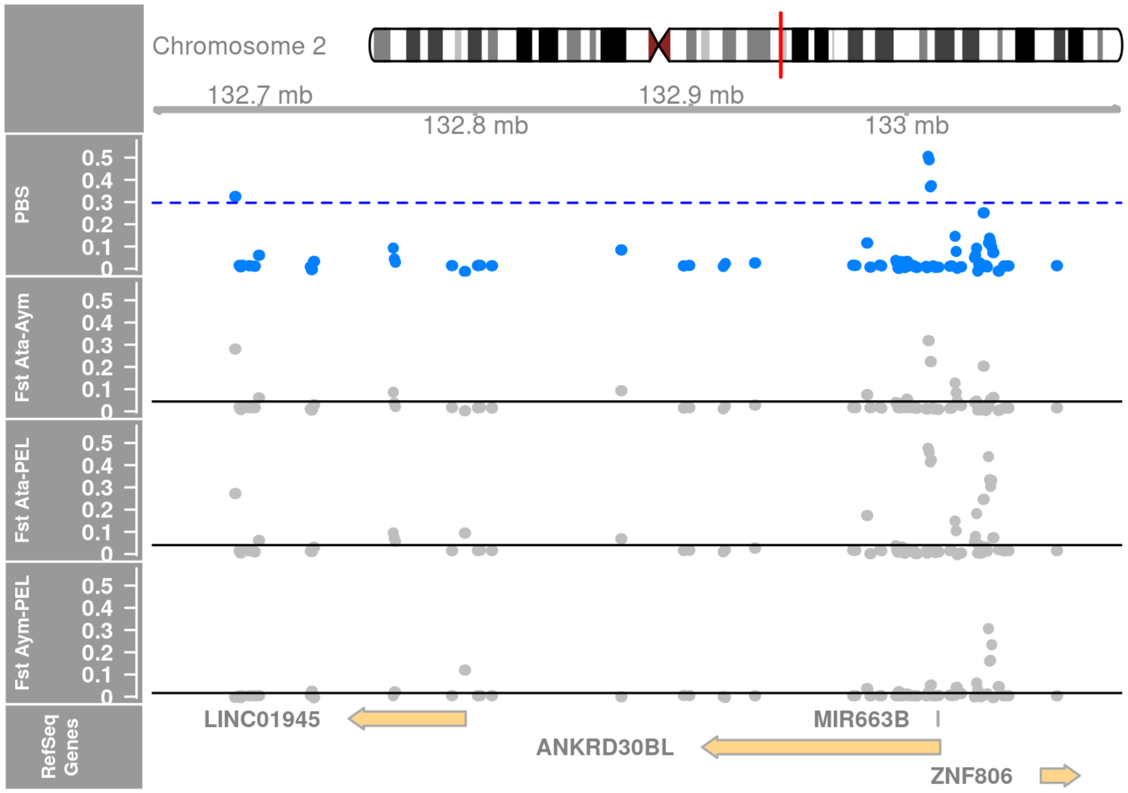

Figure S6. PBS scan peak on chromosome 2 at 143Mb. Scan performed with windows of 1kb, slide of 500bp. Dashed blue line shows 0.1 percentile of PBS. Black lines show genomewide FST values for each population pair.

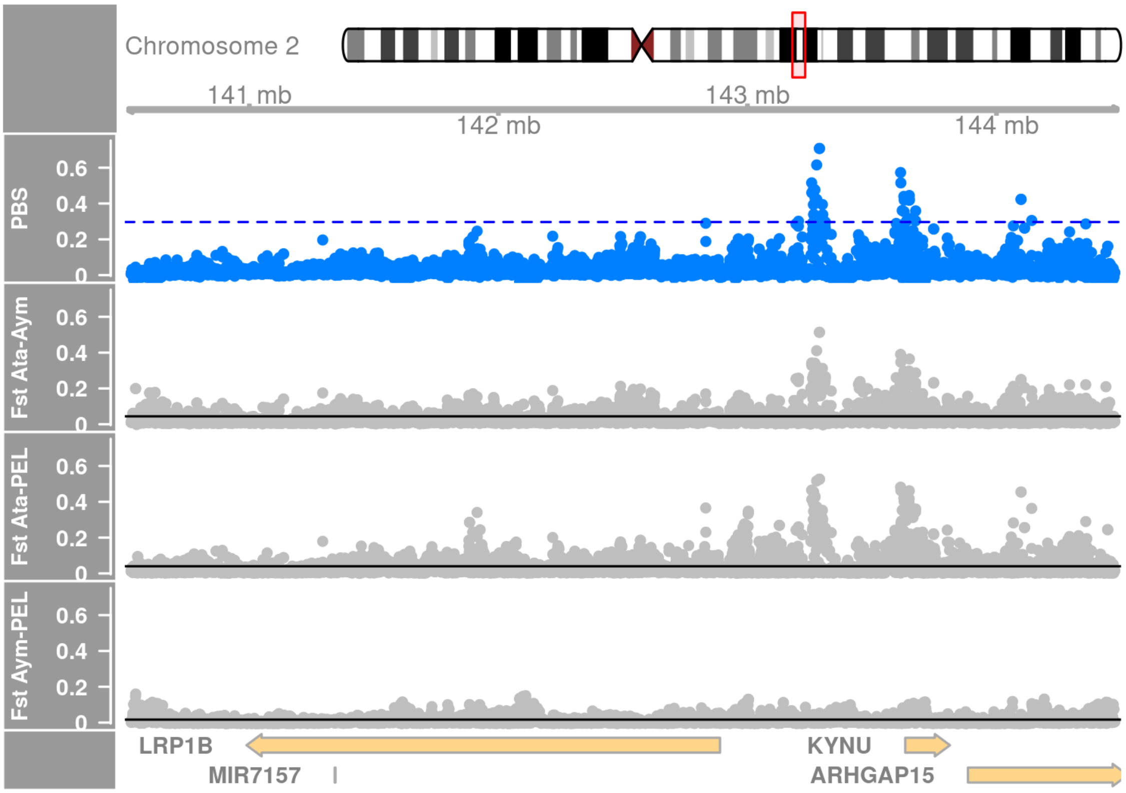

Figure S7. PBS scan peak on chromosome 1 at 155Mb. We highlight the gene *FAM189B,* which has been associated to a disease (Gaucher disease) that results from buildup of fatty substances. Scan performed with windows of 1kb, slide of 500bp. Dashed blue line shows 0.1 percentile of PBS. Black lines show genomewide FST values for each population pair.

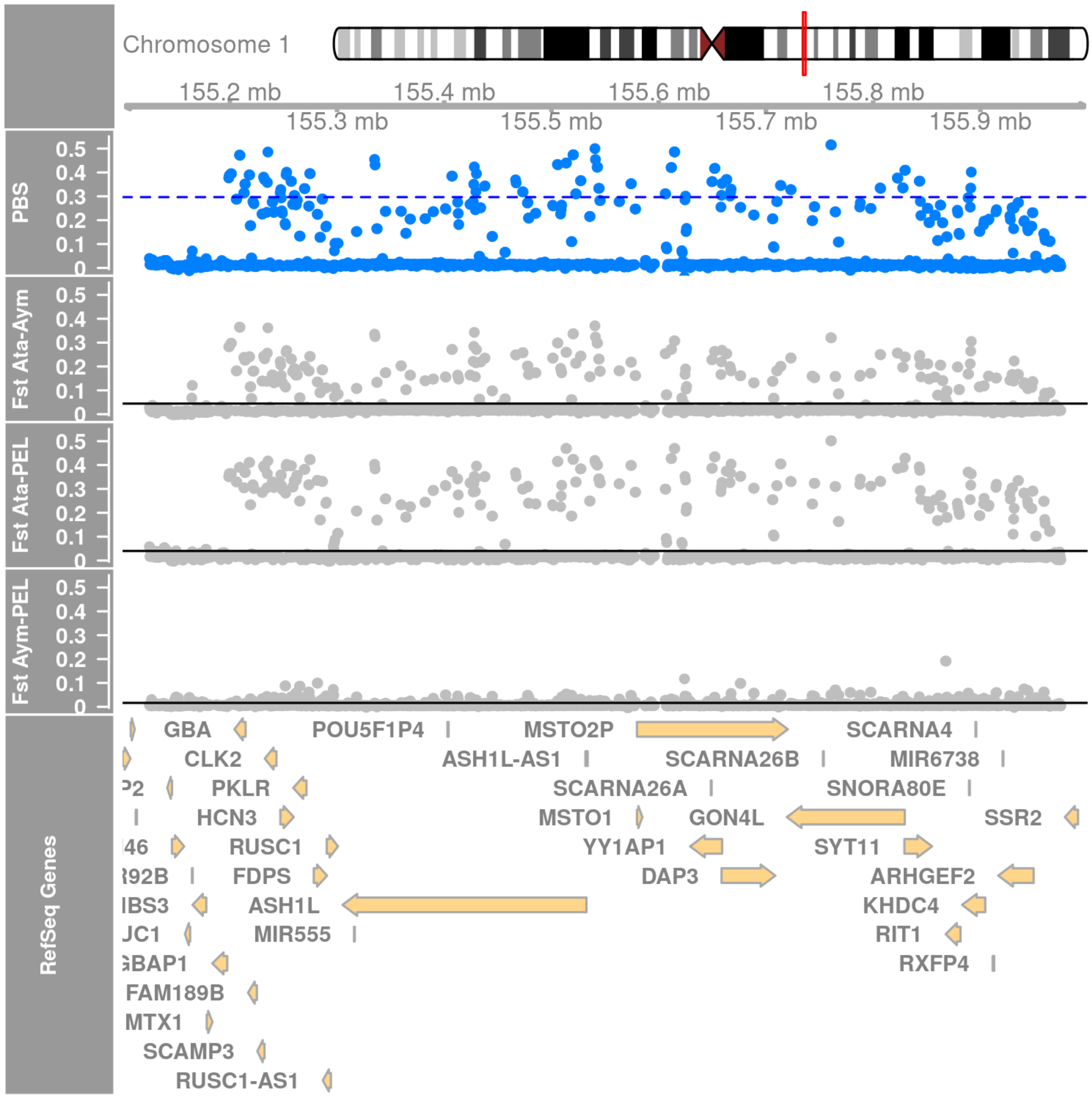

Figure S8. PBS scan peak on chromosome 1 at 26Mb. Scan performed with windows of 1kb, slide of 500bp. Dashed blue line shows 0.1 percentile of PBS. Black lines show genomewide FST values for each population pair.

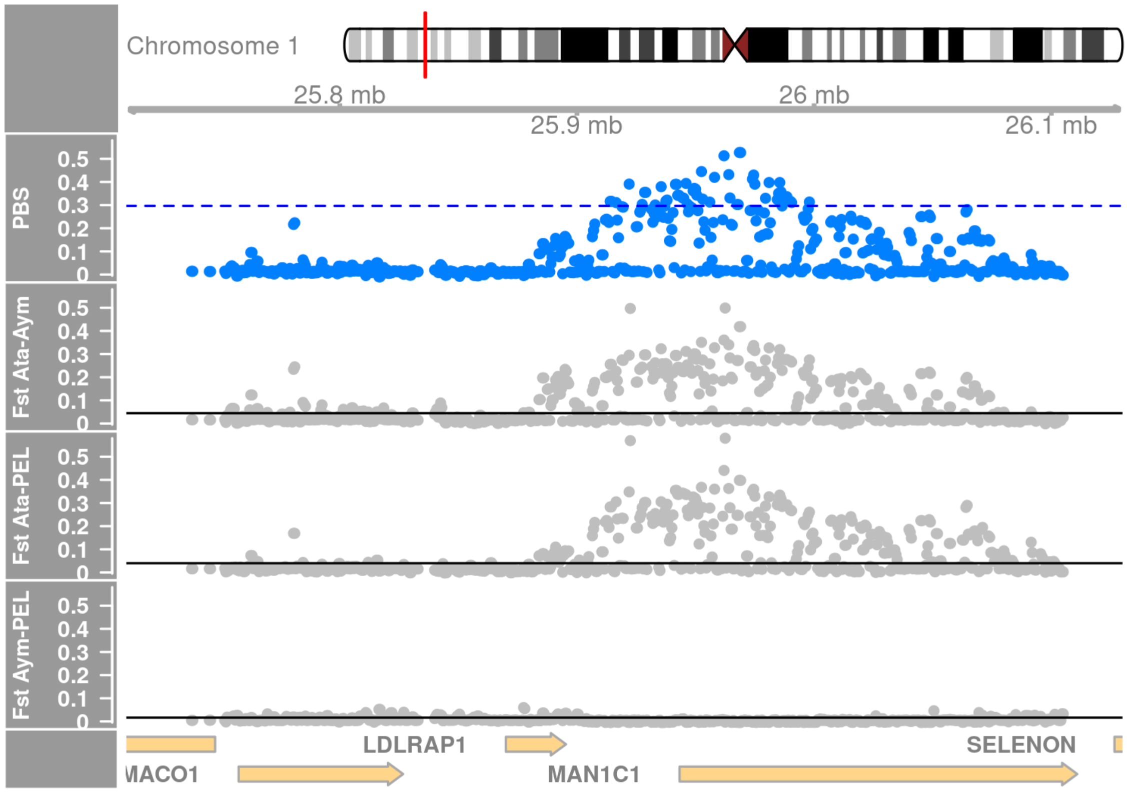

Supplement: jkaf014_Supplementary_Data [file jkaf014_supplementary_data.zip › Supplemental_Table_and_Figures_G3-2025-405654.pdf]
